# Airway and Anaesthetic Management of Adult Patients with Mucopolysaccharidoses Undergoing Cardiac Surgery

**DOI:** 10.3390/jcm13051366

**Published:** 2024-02-28

**Authors:** David Mayhew, Kenneth Palmer, Ian Wilson, Stuart Watson, Karolina M. Stepien, Petra Jenkins, Chaitanya Gadepalli

**Affiliations:** 1 Department of Anaesthesia, Liverpool Heart and Chest Hospital, Liverpool L14 3PE, UK; david.mayhew@lhch.nhs.uk (D.M.); kenneth.palmer@lhch.nhs.uk (K.P.); 2 Department of Cardiac Surgery, Liverpool Heart and Chest Hospital, Liverpool L14 3PE, UK; ian.wilson@lhch.nhs.uk; 3 Medical Physics Department, Salford Care Organisation, Northern Care Alliance NHS Foundation Trust, Manchester M6 8HD, UK; stuart.watson@nca.nhs.uk; 4 Adult Inherited Metabolic Department, Salford Care Organisation, Northern Care Alliance NHS Foundation Trust, Manchester M6 8HD, UK; karolina.stepien@nca.nhs.uk; 5 Adult Congenital Heart Disease Centre, Liverpool Heart and Chest Hospital, Liverpool L14 3PE, UK; petra.jenkins@lhch.nhs.uk; 6 Ear Nose and Throat Department, Salford Care Organisation, Northern Care Alliance NHS Foundation Trust, Manchester M6 8HD, UK; 7 School of Medical Education, The University of Manchester, Manchester M14 4PX, UK

**Keywords:** mucopolysaccharidosis, cardiac surgery, airway management

## Abstract

**Background:** Mucopolysaccharidoses (MPSs) are rare congenital lysosomal storage disorders due to a deficiency of enzymes metabolising glycosaminoglycans, leading to their accumulation in tissues. This multisystem disease often requires surgical intervention, including valvular cardiac surgery. Adult MPSs have complex airways making anaesthesia risky. **Methods:** We report novel three-dimensional (3D) modelling airway assessments and multidisciplinary peri-operative airway management. **Results:** Five MPS adults underwent cardiac surgery at the national MPS cardiac centre (type I = 4, type II = 1; ages 20, 24, 33, 35, 37 years; two males, three females). All had complex airway abnormalities. Assessments involved examination, nasendoscopy, imaging, functional studies, 3D reconstruction, virtual endoscopy, virtual reality and simulation using computerised, physical modelling. Awake oral fibre-optic intubation was achieved via airway conduit. Staged extubation was performed on the first post-operative day under laryngo-tracheoscopic guidance. The post-operative period involved chest physiotherapy and occupational therapy. All patients had safe intubation, ventilation and extubation. Four had good cardiac surgical outcomes, one (MPS type I; age 35 years) was inoperable due to endocarditis. None had post-operative airway complications. **Conclusions:** Expertise from cardiovascular-heart team, multidisciplinary airway management, use of novel techniques is vital. Traditional airway assessments are insufficient, so ENT input, radiology and computerised methods to assess and simulate the airway in 3D by collaboration with clinical engineering is essential.

## 1. Introduction

Mucopolysaccharidosis (MPS) describes a heterogenous group of rare congenital lysosomal storage disorders with a combined annual incidence of 1:22,000 [1]. The disease results from a deficiency of enzymes required to metabolise complex mucopolysaccharides called glycosaminoglycans (GAGs) [2] which in turn leads to their accumulation in soft tissues including the airways and lungs, heart valves, aorta and coronary arteries, liver and spleen, bones and the central nervous system [3,4]. This leads to multisystem morbidity and reduced longevity.

Advances in treatments such as Haemopoietic Stem Cell transplantation (HSCT) and Enzyme Replacement Therapy (ERT), alongside increasing awareness amongst health care professionals has improved the life expectancy of this patient group [5]. HSCT and ERT have been shown to reduce progression of left ventricular hypertrophy [6,7] but neither influence valvular disease progression, in particular in MPS type I, II and VI [8,9,10,11,12], which remains an unmet need [13]. One study from Japan [12] has shown HSCT to improve and stabilise valvular pathology in children with MPS II. The progressive cardiac or orthopaedic complications often need surgical intervention under anaesthetic due to multisystem disease. All adult MPS patients have some degree of airway abnormality [14], making anaesthesia for any surgical intervention complex and high risk [15,16,17,18]. The complexity of the airway is driven by a relatively large head, cervical spine instability or prior surgical fixation, cervical canal stenosis, atlantoaxial instability and deposition of GAG into the base of the tongue, temporomandibular joints and airway cartilages. This is compounded by complex ventilation requirements secondary to skeletal abnormalities as a small thoracic cage, thoracolumbar kypho-scoliosis, abnormal vertebrae, spatulate ribs and short stature [19,20,21].

Here we share our experience of airway and anaesthetic management of adult MPS patients undergoing cardiac surgery. We outline our multidisciplinary approach in pre-operative planning and simulation, a reproducible technique for safe intubation, intra operative care, staged extubation and post-operative care. We describe our detailed airway management tailored to adult MPS patients and use of novel methods developed by collaboration with clinical engineering. To the best of our knowledge, this is the first series to be presented on the airway aspects of adult MPS patients undergoing cardiac surgery.

## 2. Materials and Methods

Retrospective analysis of all adult patients with MPS who underwent valvular heart surgery in Liverpool Heart and Chest Hospital, Liverpool, United Kingdom was carried out and a literature review was undertaken as a case series. All the patients were worked up by the same cardiologist and operated on by the same cardiac surgeon. The airway assessments with three-dimensional (3D) reconstructions were carried out at Salford Care Organisation, Manchester, United Kingdom. The surgery was conducted between the years 2017 and 2023. The airway team constituted of the same cardiac anaesthetist, intensivist, and ear, nose and throat (ENT) surgeon for all the patients. Pre-operative airway planning included clinical examination, awake nasendoscopy, cross-sectional imaging using non-contrast computer tomography scan (CT scan), 3D reconstruction and virtual endoscopy (VE). Expertise from our clinical engineering department, with experience in complex airway assessment and simulation, was sought for the computerised assessment of the airways. In addition, virtual reality (VR) simulation was undertaken from CT reconstructions enabling the airway team to handle and examine the airway in the utmost detail. Physical models were made using 3D printing techniques in one patient who had two previous failed intubations, allowing for the high-fidelity simulation of intubation, and testing of airway management techniques to assess their feasibility.

Modified Mallampati grade (MP) [22] was used to assess the oral cavity. Laryngeal height and position were assessed by nasendoscopy and on CT scan. Hyomental distance (HMD) and hyomental angle (HMA) [23] were used to assess the severity of laryngeal malposition. The subglottic diameter was calculated using the CT scan and indicated the maximum size of the endotracheal tube that could be used. Tracheal anomalies such as stenosis, tortuosity, angulation and malacia were assessed using CT, 3D reconstruction and virtual endoscopy.

The software package 3D Slicer version 5.6.1 [24] was used to perform segmentation of the airway from the CT scans. Subsequent visualisation and 3D planning of use of intubation devices were carried out using the Computer Aided Design (CAD) package Fusion 360^®^ version 2.0.18460 (Autodesk, San Francisco, CA, USA), and this was also used for the design of 3D printed simulation models. VR models were created using the development environment Unity LTS 2021 (Unity Technologies, San Francisco, CA, USA).

Once the airway abnormalities had been delineated, methods to address them were planned by the multidisciplinary team.

The degree of predicted airway complexity was quantified using the Salford Adult Mucopolysaccharidosis Airway Score (SMAS) [14]. The SMAS took into account all factors from lips to lungs such as mouth opening, teeth protrusion, cervical spine mobility/stability, tongue bulkiness, modified Mallampati grade [22], thyromental distance, height of larynx, bulkiness of epiglottis/supraglottis, glottis, sub-glottic diameter, tracheomalacia/stenosis/malacia, tracheal tortuosity, FEV1% (forced expiratory volume) and FVC% (forced vital capacity). Each of these 15 parameters is scored in an ordinal score as normal, mild, moderate or severe. The minimum and maximal achievable score are 0 to 45. Appendix A shows SMAS score [14]. The comprehensive score helped to prognosticate the risk of intervention and provided guidance for health care professionals, patients and families to make an informed decision regarding cardiac surgery. Table 1 summarises the methods of airway assessment.

## 3. Results

Table 2 summarises the patient demographics, current treatment modality, associated co-morbidities and the type of cardiac surgery. All patients had a short stature and some form limited mobility. All the investigations described in Section 2 as part of the pre-operative airway assessment were carried out in all patients. However, pulmonary function tests could not be carried out in patient number four due to poor compliance. All patients had successful intubation, ventilation, extubation and successful post-operative recovery. All but one patient proceeded with planned cardiac surgery; surgery had to be abandoned in patient number four following the discovery of florid endocarditis and inflammation upon sternotomy. This patient, however, had a successful extubation and recovery and his cardiac disease was managed conservatively.

### 3.1. Observed Airway Abnormalities

**Figure 1:**
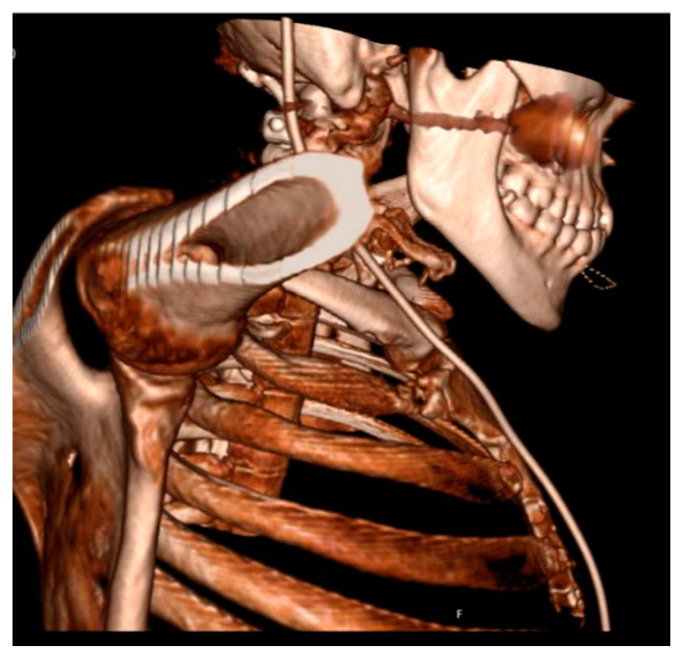
Three-dimensional reconstruction depicting large head, small spine and large jaw in patient two.

**Figure 2:**
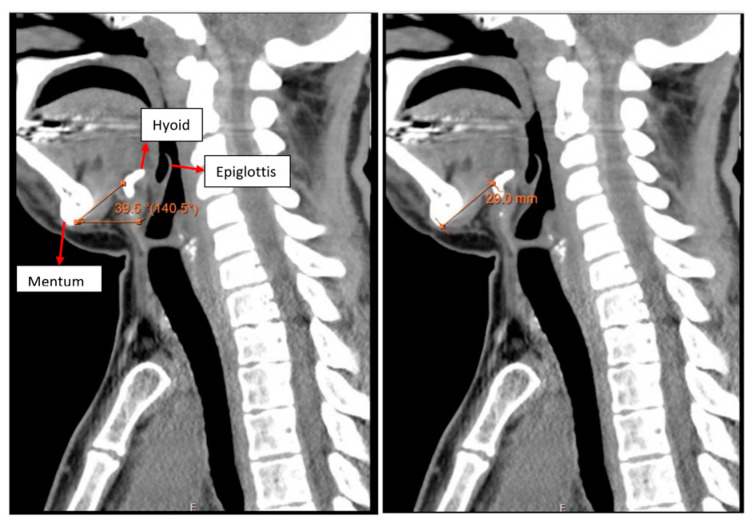
Acute hyomental angle (39.5 degrees) on the left short hyomental distance (29 mm) in patient three indicating high and anterior larynx.

**Figure 3:**
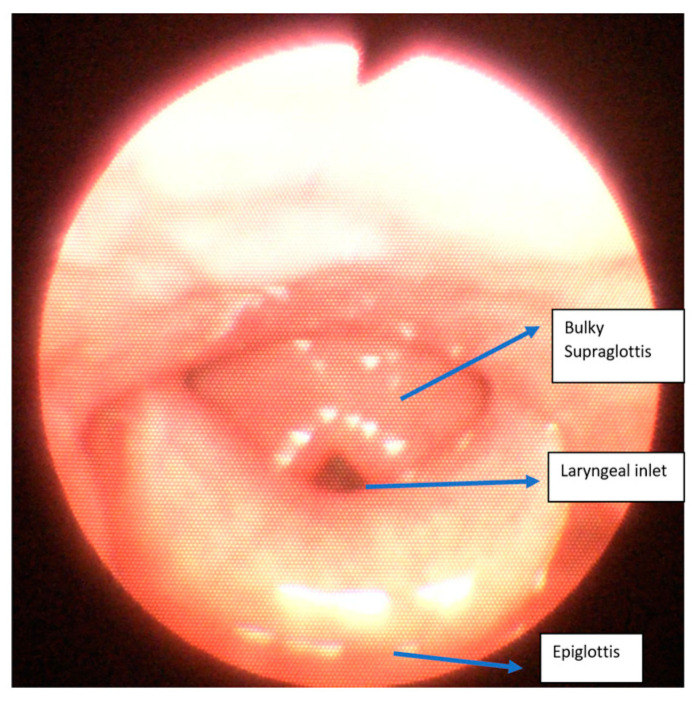
Nasendoscopy view showing large epiglottis, bulky supraglottis narrow supraglottis in patient five.

**Figure 4:**
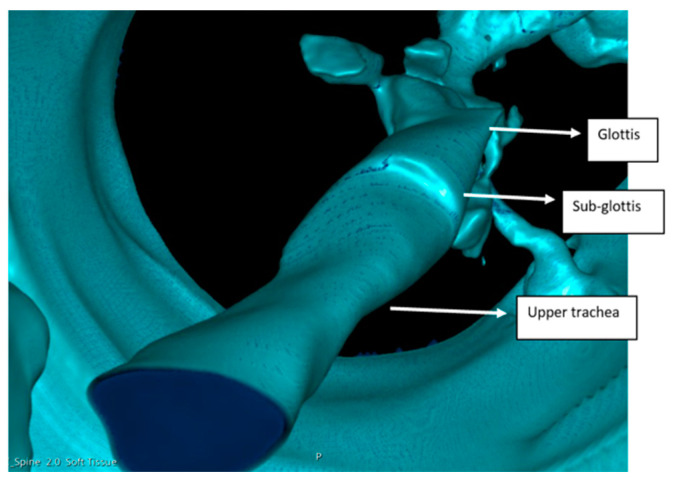
Three-dimensional (3D) reconstruction in patient four showing vertically flattened trachea below sub glottis.

**Figure 5:**
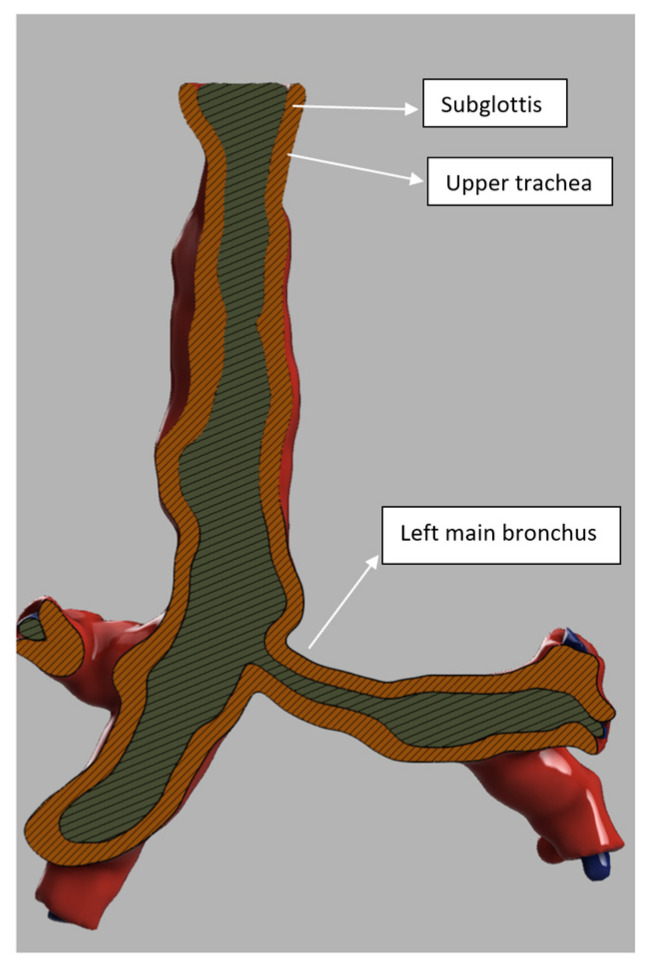
Three-dimensional (3D) reconstruction in patient four showing vertically flattened upper trachea, kinking and narrowing of left main bronchus.

All the patients had a large head, small spine, prominent teeth, limited mouth opening, high and anterior larynx and bulky anterior neck soft tissue making access to the airway challenging. Patient number two had previous spinal surgery as a child and previously needed an emergency tracheostomy due to airway compromise but was subsequently decannulated. Patient number five had two failed intubations in another trust requiring emergency airway rescue and abandonment of the procedures. All the airway abnormalities have been summarised in Table 3. Figure 1, Figure 2 and Figure 3 show various upper airway abnormalities. Figure 4 and Figure 5 shows tracheal abnormalities.

### 3.2. Simulation of Intubation

**Figure 6:**
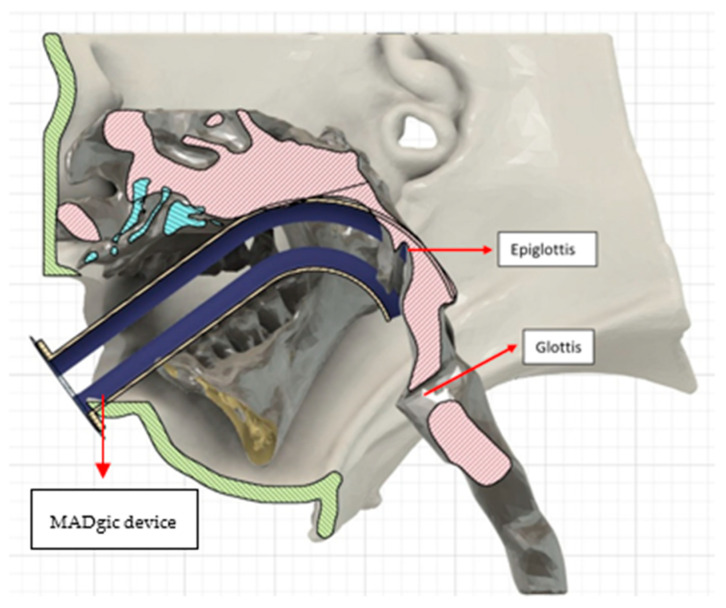
MADgic^®^ device [25] inside oral cavity, showing that the device bypasses the tongue but the epiglottis sits in the way due to high and anterior larynx.

An airway simulation of intubation was carried out in patient four and five using CAD software package Fusion 360^®^ version 2.0.18460. Computerised simulation was carried out in patient four as he did not have any previous anaesthetics, had learning difficulties and endoscopic assessments were not possible. Oral fibre-optic intubation was planned using a MADgic^®^ device (Teleflex Medical Europe Ltd., Co., Westmeath, Ireland) [25]; this device allows oral intubation under fibre-optic guidance. Simulation was carried out to see if the MADgic^®^ device [25] could be used. It was found that the epiglottis will likely sit inside the device and prevent passage of the fibre-optic scope into the glottis. Figure 6 shows CT simulation of the same.

**Figure 7:**
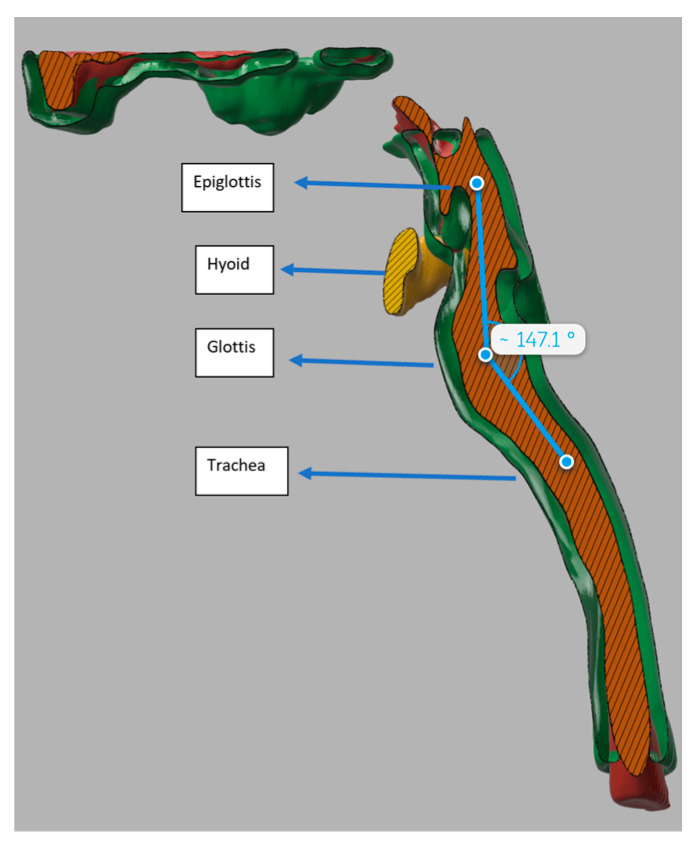
Angulated airway noted on 3D reconstruction.

**Figure 8:**
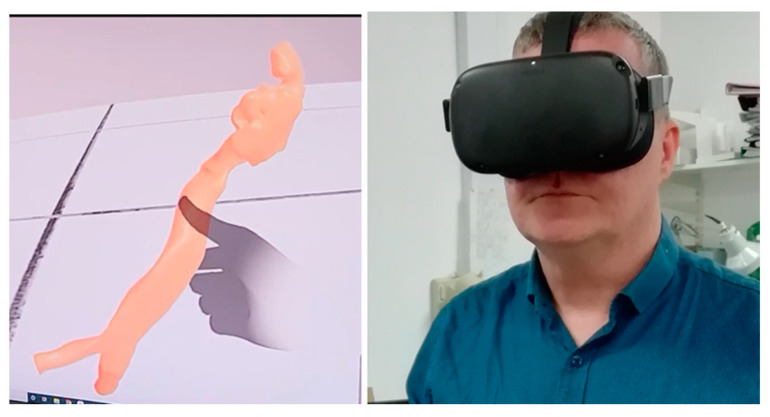
Angulated airway being manipulated utilising virtual reality.

**Figure 9:**
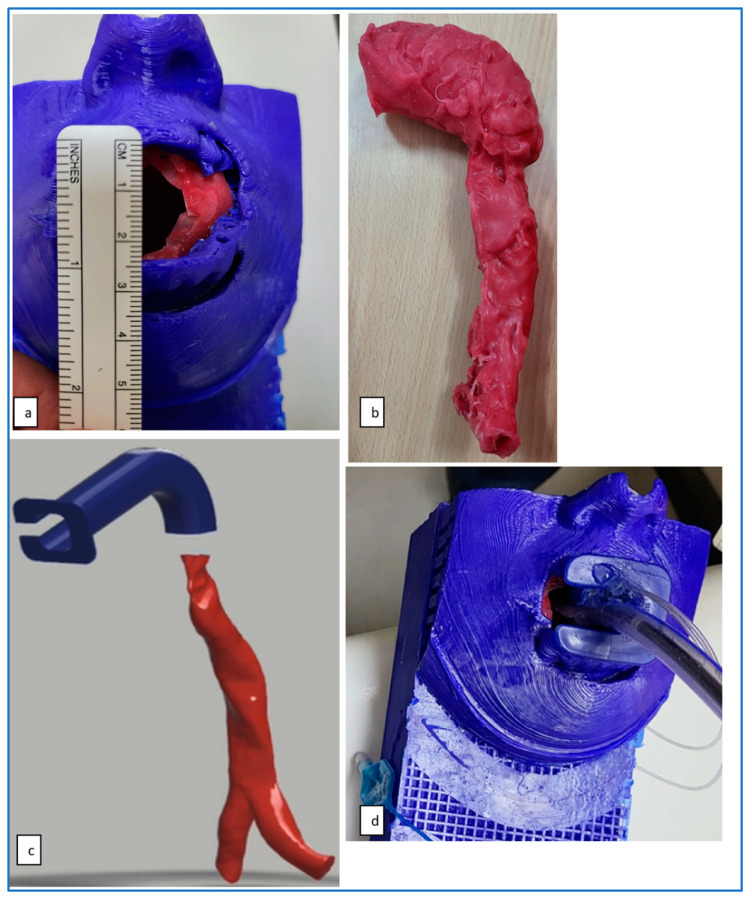
Physical three-dimensional printed model of the oropharynx (**a**), upper airway (**b**), computer simulation (**c**) and physical simulation (**d**).

In patient number five, simulation was carried out by a computer, virtual reality and physically by printing a 3D model of the airway. Patient number five had two failed intubations and failed laryngeal mask anaesthesia with difficult bag mask ventilation. The 3D reconstructions showed that the bulky larynx shown in Figure 4 had prevented bag mask ventilation and use of the laryngeal mask airway. The posteriorly angulated trachea seen in Figure 7 had prevented tracheal intubation, and railroading of the Aintree intubation catheter (Cook Medical, Bloomington, IN, USA) [26]. Figure 8 shows an angulated airway on 3D reconstruction and virtual reality. A physical model of the airway was printed to demonstrate that intubation was possible and plan management was carried out accordingly. Figure 9 shows the physically printed models and simulation on a computer.

### 3.3. Intubation

A comprehensive WHO checklist [27] with all members of the team was performed with an additional ‘airways brief’. Intubation was performed by two consultant anaesthetists, one managing the intubation, who had experience in difficult airways, and the other, administering the anaesthetic and monitoring it. Two operating department practitioners (ODP) were present and an ENT consultant was scrubbed with trays open in the operating theatre. The ODPs and ENT surgeon have experience in difficult airways and management of MPS laryngeal disease, respectively. The ENT surgeon was prepared to assist the anaesthetic team in endoscopic or front-of-neck access to the airway in the event of an upper airway crisis. A full theatre team (scrub and support staff) were in the room with airway instrument trays opened.

A myriad of equipment was made available [28], with a selection of endotracheal tubes of both adult and paediatric sizes, video laryngoscopes, flexible endoscopy for awake oral/nasal intubation with both adult and paediatric scopes, small suction catheters for smaller tubes, cricothyroid puncture set, tracheostomy instrument tray with various sizes of tracheostomy tubes, a rigid laryngo-tracheoscopy tray with a Hopkins rod telescope and a MADgic^®^ device [25] for oral fibre-optic intubation. High-flow nasal oxygen is administered from arrival to facilitate an Trans Nasal Humidified Rapid Insufflation Ventilation Exchange (THRIVE) [29].

Routine monitoring such as ECG, saturations and bispectral monitoring (BIS) were attached [30]. Due to limb contractures, it was not possible to site arterial access awake in these cases. The airway procedure was commenced by spraying the oropharynx with 4% xylocaine. High-flow nasal oxygen was commenced and oxygen flow gradually increased. Target-controlled remifentanil was administered to produce conscious sedation. The MADgic^®^ device [25] device was gently passed in the oral cavity and further airway anaesthesia was provided by aerosolization of 1% lidocaine onto the larynx. A fibre-optic scope was passed into the trachea via the MADgic^®^ device [25] and a size 7.0 Portex Blueline (Smiths Medical, Plymouth, MN, USA) cuffed endotracheal tube was railroaded into the trachea. Following intubation, anaesthesia was induced with propofol, and muscle paralysis was achieved with rocuronium. Ventilation was then commenced. Intravenous dexamethasone 6.6 mg every 8 h was commenced from theatre.

### 3.4. Extubation and Recovery from Anaesthesia

Following cardiac surgery, the patients were managed in the cardiac intensive care unit. Extubation was carried out on post-operative day one in a staged manner during normal working hours in the cardiac intensive care unit with an intensivist, anaesthetist, ENT surgeon and operating department practitioner present. The patients were sat semi upright to reduce the splinting of the diaphragm and high-flow nasal oxygen was applied before extubation and continued into the post-extubation phase. A difficult airway trolley was in the bedspace, and the team was prepared for immediate re-intubation should the need arise, with access to emergency drugs and anaesthetic equipment. Adrenaline 1:1000 1 mL was nebulised in 3 mL of 0.9% saline to reduce any airway oedema. Pre-extubation tracheoscopy was carried out to rule out airway injury or oedema secondary to intubation and to clear all secretions. Then, the cuff of the endotracheal tube was deflated to assess for air leak. The presence of an air leak confirmed an adequate airway calibre with manageable oedema. An Aintree intubation catheter [26] was placed inside the endo tracheal tube under tracheoscopic guidance and the endo tracheal tube was removed. Secretions were cleared again and the catheter was left in situ for a period of 20 min whilst the patient regained full airway control. The fibrescope was again inserted into the Aintree intubation catheter [26], and both were removed under tracheoscopy guidance to confirm no airway trauma. Adrenaline nebulisation was given every 6 h.

High-flow nasal oxygen was gradually weaned and the patients were commenced on saline nebulisation and aggressive chest physiotherapy. This ensured optimal secretion clearance and helped to splint open the upper and lower airways. Provision to access a cardiac intensivist with a special interest in difficult airways and an ENT surgeon was made.

## 4. Discussion

We have demonstrated that with a careful holistic assessment and airway planning by traditional and advanced methods, safe outcomes are possible and reproducible. Management of airways in adults with MPS disorders is complex due to multisystem involvement. The advances in treatments and increasing awareness has improved the life expectancy of this group of patients [5]. Despite these, valvular heart disease continues to progress and ultimately requires surgical intervention [8,9,10,11,12]. Due to complex airway anatomy, any surgical intervention in these patients is preferably performed under loco-regional anaesthesia; however, this is not possible for cardiac surgery. Anaesthetic interventions in these patients are very high risk and may have unfavourable outcomes [26,31,32]. Due to a variety of airway abnormalities [14], oral intubation can be extremely difficult. The difficulties derive from poor access, such as limited mouth opening, prominent teeth, high Mallampati grade [22], large tongue and a small jaw. Often these patients have a short neck, large head and spinal pathology [19,20,21], making any form of manipulation of the neck risky. These patients also have a high and anterior larynx [14,23], making visualisation of the laryngeal inlet extremely difficult.

**Figure 10:**
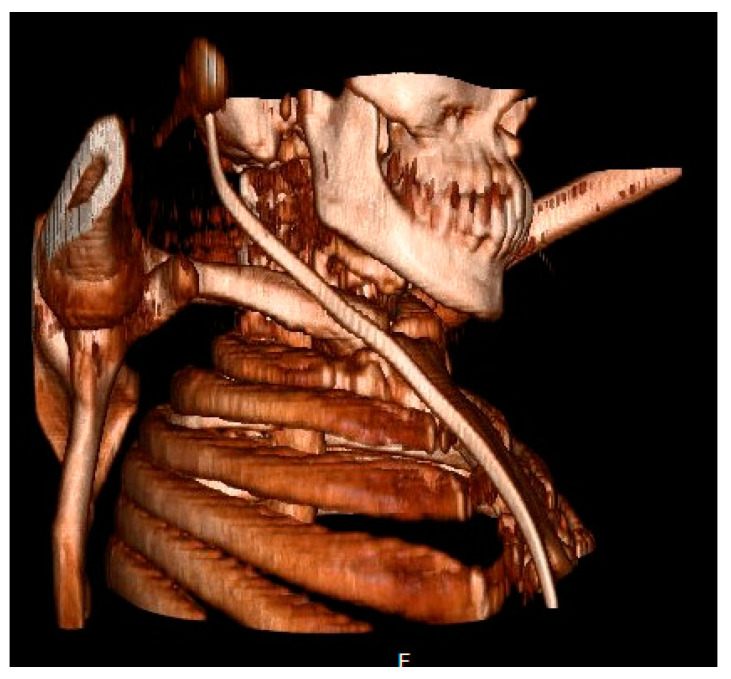
Three-dimensional reconstruction of the chest wall in an adult MPS1 with large spatulate ribs restricting lung expansion. NB: the presence of pre-existing ventriculo-peritoneal shunt.

It is essential to have a structured airway management plan that could be modified as per the Difficult Airway Society (DAS) UK guidelines [33]. The airway plan should include methods to address the feasibility of each step of intubation, supraglottic airway device, bag mask ventilation and front-of-neck approach. We have found the peri-operative use of high-flow nasal oxygen such as THRIVE [29] to be very useful during induction and extubation, and it provides a degree of safety for the procedure; we would recommend it for all adult MPS patients in the surgical setting, based on our personal experience in treating adult MPS patients. Further research into this aspect will be very helpful. In our experience, we have found that the use of supraglottic airway devices such as the laryngeal mask airways to be unhelpful when the tongue is large and the supraglottis is bulky, as commonly found in adult MPS [14]. Based on our personal experience, tracheal tortuosity, airway stenosis and tracheomalacia make intubation more challenging. Whilst very small endo tracheal tubes could be passed and secured in the airway, in adults this will result in high-ventilatory pressures, reduced tidal volumes and secretion retention, making their use suboptimal. The size of the tube is best determined by measuring the airway calibre at several levels of trachea and the sub-glottis on a CT scan. Despite securing the airway, ventilation in adult MPS can be difficult due to poor lung function [34,35] and skeletal restriction. The skeletal abnormality restricting lung function in an MPS patient is shown in Figure 10.

All the five patients in our cohort received detailed airway assessments, which included history, clinical examination, awake nasendoscopy (apart from patient four due to learning difficulties), cross-sectional imaging with additional assessment and reconstruction in 3D using CAD, virtual endoscopy, virtual reality and physical simulation of the airway. Additionally, all patients’ airway risks were quantified using the SMAS [14]. These methods in addition to routine airway assessments have helped us to understand the airway abnormalities and plan their management. The quantification of the airway issues prior to the surgery helped us to address the risk-to-benefit balance of airway intervention for cardiac surgery. This helped us to make informed decisions; we also use the images and measurements during discussion with patients and family members.

Due to the dense anticoagulation required during cardiopulmonary bypass, nasal intubation was not considered. Even minor nasal mucosal trauma can result in life-threatening epistaxis. Trauma to nasal mucosa by instrumentation additionally will make intubation more challenging and adds unnecessary risk to the intervention. We have found awake oral fibre-optic intubation to be the optimal technique. In all cases, this was performed using the MADgic^®^ device [25]. Sjøgren et al. [32] recommend spontaneous respiration until the patient has been intubated, and this is easily facilitated with target-controlled remifentanil infusion. The MADgic^®^ device [25] obviates many problems associated with accessing the supraglottis due to a large tongue, poor cervical spine extension, high anterior larynx and bulky supraglottis. The MADgic^®^ device can be used for the atomisation of local anaesthetic and oxygenation [25], but we chose to use a laryngoscopic approach and THRIVE for these cases. Yadav et al. [36] noted that airway nerve blocks were superior to atomisation; we have however found use of topical 4% xylocaine spray adequate to anaesthetise the larynx and pharynx, without the additional risk of bleeding from systemic heparinisation following a block. We also believe that nerve blocks in a short MPS neck with redundant soft tissue will be extremely challenging via injection, both via anatomical and ultrasound guided routes.

There are other types of airway conduit devices for awake oral fibre-optic intubation such as Ovassapian airway^®^, modified William’s airway^®^, modified Guedel airway^®^ and Berman airway^®^. Greenland et al. [37] found Williams^®^ and Berman^®^ superior to Ovassapian^®^ airway. Khattab et al. [38] found the modified Williams airway^®^ most useful when compared to the modified Guedel^®^ airway and LMA MADgic^®^ airway. In our experience, we have found the use of the MADgic^®^ device to be most readily available, and the most appropriate in terms of angulation and size in adult MPS patients. In the future, customised airway conduits by CAD and 3D printing may be an option. We have built a custom airway conduit device by 3D design in our adult MPS patients and we are yet to publish our results.

Following surgery, a sleep apnoea-like picture could be seen due to bulky upper airways and tracheomalacia. Adult MPS patients are inherently prone to obstructive sleep apnoea syndrome [39,40,41]. In the post-extubation period, this will be exacerbated due to residual anaesthesia and airway oedema following instrumentation and intubation. We managed this by judicious use of opiates, staged extubation, high-flow nasal oxygen, regular steroids for the first 72 h, nebulised adrenaline for the first 24 h and sitting the patient upright. All of our patients are kept in intensive care for at least 24 h following extubation. Retention of secretions is a recognised problem following extubation; we managed this by chest physiotherapy, mucolytic administration and normal saline nebulisation. Sjøgren et al. [32] addressed the problems of secretion management similarly and mentions the importance of positive end-expiratory pressure with a low threshold for antibiotics where necessary.

Mucopolysaccharidosis is a very rare condition with a combined annual incidence of 1:22,000 [1]; there are not many patients who make it to adulthood. Amongst those who have reached adulthood, not all require cardiac surgery. This paper specifically focuses on those needing cardiac surgery. Hence, the study size is small; however, to the best of our knowledge, this is the largest series reported. Due to the limited size, statistical analysis or comparison with a control group is not possible. Further studies with a larger group of patients in both cardiac and non-cardiac conditions will be useful to assess long-term outcomes. A discussion of patient experiences will also be useful to understand their journey. Our methods are reliant on advanced computerised-aided design from the expertise of clinical engineering; this expertise may not be available at all tertiary centres.

## 5. Conclusions

The multidisciplinary approach is essential to make a safe airway management plan for adults with MPS disorders and related conditions as part of the cardiac surgery work-up. The role of clinical engineering is important in the evaluation of precise airway dimensions and understanding airflow dynamics. Our clinical outcomes of intubation, ventilation, extubation and recovery have been favourable purely due to this structured approach. We recommend a holistic approach through specialist input from an airway ENT specialist, cardiac anaesthetist, clinical engineer, metabolic medicine physician, cardiac physician and cardiac surgeon for favourable outcomes.

## 6. Patents

The techniques of 3D reconstruction have been developed by the 3DSPIN unit (3D surgical planning and intervention) at Salford Care Organisation, Manchester, UK

## Figures and Tables

**Table 1:** Airway assessment methods and methods of assessments of each airway parameter.

**Assessment methods**	- Clinical examination - Nasendoscopy - Cross-sectional imaging - Three-dimensional (3D) reconstruction - Virtual endoscopy - Virtual reality - Printing 3D airway models - Simulation of intubation by printing and in - computer
**Parameters Assessed**	**Method of Assessment**
Mouth opening, teeth protrusion, Mallampati grade, cervical spine mobility/stability	Clinical examination
Tongue bulkiness	Clinical examination and CT scan
Laryngeal height and position	Nasendoscopy, CT scan
Subglottic diameter	CT scan
Tracheal abnormalities	CT scan, 3D reconstruction, virtual endoscopy, virtual reality
Pulmonary functions	Spirometry
Simulation of intubation	3D printed models, computerised methods, virtual reality

**Table 2:** Demography of the adult MPS patients undergoing cardiac surgery. Pt—patient number, MPS—mucopolysaccharidosis, M—male, F—female, ERT—enzyme replacement therapy, Kg—kilograms, cm—centimetres, BMI—body mass index in kg/m^2^.

Pt	MPS Type	Sex	Age in Years	Weight Height BMI	Current MPS Treatment	Associated Problems	Cardiac Surgery
1	II	M	20	63.4 kg 157.4 cm 25.6	ERT	Trismus, epilepsy, cervical canal stenosis, carpal tunnel, tibial plates	Aortic valve replacement
2	I	F	37	68 kg 167.6 cm 24.4	None	Poor mobility due to arthritis, cervical laminectomy and fusion with post-operative respiratory arrest needing tracheostomy, hip replacement, ventriculoperitoneal shunt	Aortic and mitral valve replacement, with tricuspid repair
3	I	F	24	58.9 kg 163 cm 22.1	ERT	Poor mobility due to arthritis, myelomalacia cervical spine, cervical foraminal stenosis at C3/C4, C4/C5	Aortic valve replacement
4	I	M	35	44 kg 148 cm 20.1	None	Learning difficulties	Aortic root abscess—not operated
5	I	F	33	49 kg 153 cm 20.9	ERT	Poor vision in one eye, poor mobility, cervical spine stiffness, carpal tunnel	Mitral valve replacement

**Table 3:** Summary of the airway abnormalities in all the five patients.

Pt	MPS Type	Sex	Airway Abnormalities	FEV1%	FVC%	SMAS
1	II	M	Mouth opening 2 cm, MP grade 3, small spine, prominent incisors, cervical canal stenosis, high anterior larynx, bulky supraglottis, flattening of mid and lower trachea	82	88	17/45
2	I	F	Mouth opening 3 cm, small fixed spine, 30–60 degrees spine flexion extension, MP grade 3, prominent incisors, bulky tongue, anterior larynx, mild tracheal stenosis from previous tracheostomy	50	55	18/45
3	I	F	Mouth opening 2.5 cm, myelomalacia of cervical spine, small spine, large tongue, hypognathia, MP grade 3, high anterior larynx, large epiglottis, mild tracheomalacia	59	60	10/45
4	I	M	Large head, small spine, mouth opening 3 cm, large bulky tongue, high anterior larynx, bulky supraglottis, upper tracheal vertical flattening with narrowing, left main bronchus narrowing, deep cervical trachea, learning difficulties	Not performed	Not performed	14/30
5	I	F	Short spine, small jaw, bulky tongue, bulky supraglottis, high and anterior larynx, angulated trachea, tracheomalacia, two previous failed intubations	38	38	26/45

Pt—patient number, M—male, F—female, MP—modified Mallampati grade [22], FEV1—forced expiratory volume, FVC—forced vital capacity, SMAS—Salford Adult Mucopolysaccharidosis Airway Score (score of patients airway/maximum possible score).

## Data Availability

All the data relevant to the paper has been presented. Any further information may be requested from the corresponding author. No personal identifiable information will be provided.

## Appendix A

The Salford Mucopolysaccharidosis Airway Score (SMAS) [14] includes all factors from the lips to the lungs such as mouth opening, teeth protrusion, cervical spine mobility/stability, tongue bulkiness, modified Mallampati grade [22], thyromental distance, height of larynx, bulkiness of epiglottis/supraglottis, glottis, sub-glottic diameter, tracheomalacia/stenosis/malacia, tracheal tortuosity, FEV1% (forced expiratory volume), FVC%. (forced vital capacity). Each of these 15 parameters is scored in an ordinal score as normal, mild, moderate or severe. The minimum and maximal achievable score ranges are from 0 to 45.

**S. No.**	**Parameter**	**Measure**	**Score**	**Final Score**
	MPS Type			
1	Mouth opening	>5 cm	0	
		4–5 cm	1	
		3–4 cm	2	
		<3 cm	3	
2	Teeth protrusion on clinical exam and scans	Non-protruding	0	
		Mild	1	
		Moderate	2	
		Severe	3	
3	Cervical spine mobility, stability	Unrestricted	0	
		60–90 degrees flexion	1	
		30–60 degrees flexion	2	
		<30 degrees or unstable	3	
4	Tongue bulkiness on examination and scan	Normal	0	
		Mild (filling less than 1/3 of floor mouth)	1	
		Moderate (filling 1/3 to 1/2 of oral cavity)	2	
		Severe (filling more than 1/2 of oral cavity)	3	
5	Modified Mallampati grade [22]	1	0	
		2	1	
		3	2	
		4	3	
6	Thyromental distance	>6 cm	0	
		5–6 cm	1	
		4–5 cm	2	
		<4 cm	3	
7	Larynx height epiglottis to soft palate	>4 cm	0	
		3–4 cm	1	
		2–3 cm	2	
		<2 cm	3	
8	Epiglottis bulkiness	Normal (filling less than 1/3 of oropharynx)	0	
		Mild (filling 1/3 to 1/2 of oropharynx)	1	
		Moderate (filling 1/2 to complete oropharynx)	2	
		Severe (Filling entire oropharynx)	3	
9	Supraglottis bulkiness	Normal (filling less than 1/3 of laryngopharynx)	0	
		Mild (filling 1/3 to 1/2 of laryngopharynx)	1	
		Moderate (filling ½ to complete laryngopharynx)	2	
		Severe (filling entire oropharynx)	3	
10	Glottis bulkiness	Normal (filling less than 1/3 of glottis)	0	
		Mild (filling 1/3 to 1/2 of glottis)	1	
		Moderate (filling 1/2 to complete glottis)	2	
		Severe (filling entire glottis)	3	
11	Subglottis diameter at cricoid level	>7 mm	0	
		6–7 mm	1	
		5–6 mm	2	
		<5 mm	3	
12	Tracheomalacia or tracheal stenosis (degree of narrowing)	No narrowing	0	
		50–75% lumen narrowing	1	
		75–99% lumen narrowing	2	
		100% lumen narrowing	3	
13	Tracheal tortuosity	None	0	
		present	3	
14	FEV1%	>80%	0	
		60–79%	1	
		40–59%	2	
		<40%	3	
15	FVC%	>80%	0	
		60–79%	1	
		40–59%	2	
		<40%	3	
